# Supplementary material for: A Simple Vortex-Based Method for the Generation of High-Throughput Spherical Micro- and Nanohydrogels
Source: Int J Mol Sci. 2025 Jun 30;26(13):6300. doi: 10.3390/ijms26136300 (PMC12250470; doi:10.3390/ijms26136300)
Supplement: Supplementary file 1 [file ijms-26-06300-s001.zip › ijms-3693701 supplementary.pdf]

Supporting information

Table S1. Obtained means, coefficient of variation (C.V.) and polydispersity indexes (PDI) the measured micro hydrogels presented in Figure 2 and 3.

Figure 2	Conditions	Mean (μm)	Polydispersity index (PDI)	Coefficient of variation (C.V.)
A. Surfactant concentration (%)	1	4.89	0.50	0.71
	2	4.16	0.45	0.67
	5	4.17	0.23	0.48
	10	3.32	0.14	0.37
B. Eppendorf® size (mL)	0.5	4.58	0.35	0.59
	1.5	4.39	0.11	0.34
	5	3.25	0.12	0.34
C. Vortex speed (rpm)	1200	8.19	0.41	0.64
	1600	8.70	0.27	0.52
	2000	6.16	0.32	0.56
	2400	4.89	0.21	0.46
	3000	4.25	0.14	0.38
D. Vortex duration (s)	30	4.46	0.37	0.61
	60	3.97	0.26	0.51
	120	3.58	0.17	0.41
	240	3.32	0.16	0.40
	360	3.32	0.14	0.37
E. Oil-to-polymer ratio (uL)	50-50	5.90	0.29	0.54
	100-50	5.62	0.15	0.39
	200-50	4.17	0.16	0.40
	400-50	3.72	0.20	0.45
	600-50	3.95	0.20	0.44
	800-50	4.52	0.23	0.48
F. Oil-to-polymer ratio (uL) with consistent oil	250-50	3.41	0.19	0.43
	250-100	3.32	0.16	0.40
	250-200	3.66	0.13	0.37

Figure 3	Conditions	Mean (μm)	Polydispersity index (PDI)	Coefficient of variation (C.V.)
A. Polymer MW	575	5.07	0.07	0.26
	700	5.22	0.29	0.54
B. Pulsed vortexing (s)	0	3.82	0.15	0.39
	5	3.57	0.12	0.35
	10	3.49	0.14	0.37
	30	3.23	0.15	0.38
C. Reproducibility	Batch 1	5.07	0.07	0.26
	Batch 2	4.91	0.09	0.31
	Batch 3	5.02	0.08	0.28

Table S2. Dynamic light (DLS) data of nanogels purified via centrifugation presented in Figure 4. Hydrodynamic diameter and polydispersity index (PDI) are shown. The values highlighted in gray represent independent batches in which the same variables were tested, yielding similar size and PDI values, thereby demonstrating reproducibility.

Variable	Conditions	Hydrodynamic diameter (nm)	Polydispersity index (PDI)
Surfactant concentration (%)	1	157	0.08
	5	149	0.08
	10	161	0.05
Cross-link density (w/w%)	20	179	0.13
	30	161	0.06
	40	170	0.06
Oil-to-polymer ratio (µL) w consistent polymer	50-50	204	0.13
	100-50	177	0.06
	200-50	180	0.06
	400-50	161	0.05
	800-50	165	0.05
PEGDA MW	575	161	0.05
	700	149	0.04
Vortex speed (RPM)	2000	169	0.07
	2400	165	0.06
	3000	**156**	**0.05**
Vortex duration (s)	30	137	0.11
	60	142	0.11
	120	146	0.09
	240	**161**	**0.05**
	480	165	0.05
Oil-to-polymer ratio (µL) w consistent oil	400-40	154	0.05
	400-30	161	0.04
	400-20	136	0.09
	400-10	126	0.20
Oil viscosity	n-Hexadecane	**160**	**0.06**
	Squalene	207	0.09

Table S3. Concentrations of different hydrogel populations. Concentrations are presented here as particles per mL.

Variable	Conditions	Microgels (≥ 5 μm)	Microgels (< 5 μm)	Nanohydrogels (< 1 μm)	Total particle count
Surfactant concentration (%)	1	6.09E+07	2.99E+09	1.04E+11	1.10E+11
	5	5.87E+07	2.41E+09	3.52E+10	4.01E+10
	10	2.18E+05	2.03E+09	2.26E+11	2.30E+11
Cross-link density (w/w%)	20	3.46E+04	2.82E+08	1.18E+11	1.10E+11
	30	1.26E+05	2.13E+09	1.75E+11	4.01E+10
	40	4.10E+07	4.61E+09	1.73E+11	2.30E+11
Oil-to-polymer ratio (μL) w consistent polymer	50-50	9.48E+07	1.52E+08	4.73E+10	4.78E+10
	100-50	7.86E+07	6.06E+08	2.18E+11	2.19E+11
	200-50	8.88E+07	1.43E+09	2.93E+11	2.96E+11
	400-50	1.55E+05	5.06E+08	2.87E+11	2.88E+11
	800-50	3.82E+05	7.91E+08	1.12E+11	1.14E+11
PEGDA MW	575	2.18E+05	2.03E+09	2.26E+11	2.88E+11
	700	5.14E+07	1.84E+09	1.37E+11	1.41E+11
Vortex speed (RPM)	2000	6.04E+07	6.67E+08	2.68E+11	2.70E+11
	2400	2.03E+07	1.20E+09	2.20E+11	2.22E+11
	3000	2.12E+05	1.88E+09	1.43E+11	1.47E+11
Vortex duration (s)	30	8.11E+07	9.51E+08	1.37E+11	1.39E+11
	60	4.43E+07	1.66E+09	1.90E+11	1.94E+11
	120	9.52E+06	1.89E+09	1.90E+11	1.94E+11
	240	2.18E+05	2.03E+09	2.26E+11	2.88E+11
	480	0.00E+00	2.52E+09	2.34E+11	2.39E+11
Oil-to-polymer ratio (μL) w consistent oil	400-40	2.22E+05	1.20E+09	1.91E+11	1.93E+11
	400-30	2.83E+05	5.96E+08	1.40E+11	1.42E+11
	400-20	6.00E+05	2.23E+08	6.93E+10	6.97E+10
	400-10	6.67E+05	1.31E+07	7.29E+09	7.32E+09
Oil viscosity	n-Hexadecane	2.18E+05	2.03E+09	2.26E+11	2.30E+11
	Squalene	3.45E+06	2.44E+09	3.10E+11	3.15E+11

Table S4. Volume fractions (%) of the formed micro and nano hydrogels in each separate condition.

Variable	Conditions	Microgel volume fraction	Nanogel volume fraction
Surfactant concentration (%)	1	93.4%	6.6%
	5	84.5%	15.5%
	10	84.0%	16.0%
Cross-link density (w/w%)	20	52.2%	47.8%
	30	85.5%	14.5%
	40	80.4%	19.6%
Oil-to-polymer ratio (μL)	50-50	89.2%	10.8%
	100-50	72.8%	27.2%
	200-50	70.6%	29.4%
	400-50	85.8%	14.2%
	800-50	89.1%	10.9%
PEGDA MW	575	85.8%	15.3%
	700	94.2%	5.8%
Vortex speed (RPM)	2000	88.1%	11.9%
	2400	91.6%	8.4%
	3000	88.0%	12.0%
Vortex duration (s)	30	91.3%	8.7%
	60	88.0%	12.0%
	120	92.5%	7.5%
	240	85.8%	14.2%
	480	77.1%	22.9%
Oil-to-polymer ratio (μL) w consistent oil	400-40	88.1%	11.9%
	400-30	52.0%	48.0%
	400-20	64.1%	35.9%
	400-10	84.6%	15.4%
Oil viscosity	n-Hexadecane	80.5%	19.5%
	Squalene	48.0%	52.0%

Table S5. Viscosity measurements of the used polymer solutions.

Solution	Concentration (% w/w)	Viscosity (cP)
PEGDA 575	20	2.28
	30	3.38
	40	5.45
PEGDA 700	30	4.30

Table S6. Mass based swelling ratio (g/g) of the hydrogels at different crosslink densities.

Solution	Concentration (% w/w)	Swelling ratio (g/g)
PEGDA 575	20	5.09 ± 0.01
	30	3.59 ± 0.02
	40	2.55 ± 0.06
PEGDA 700	30	3.34 ± 0.18

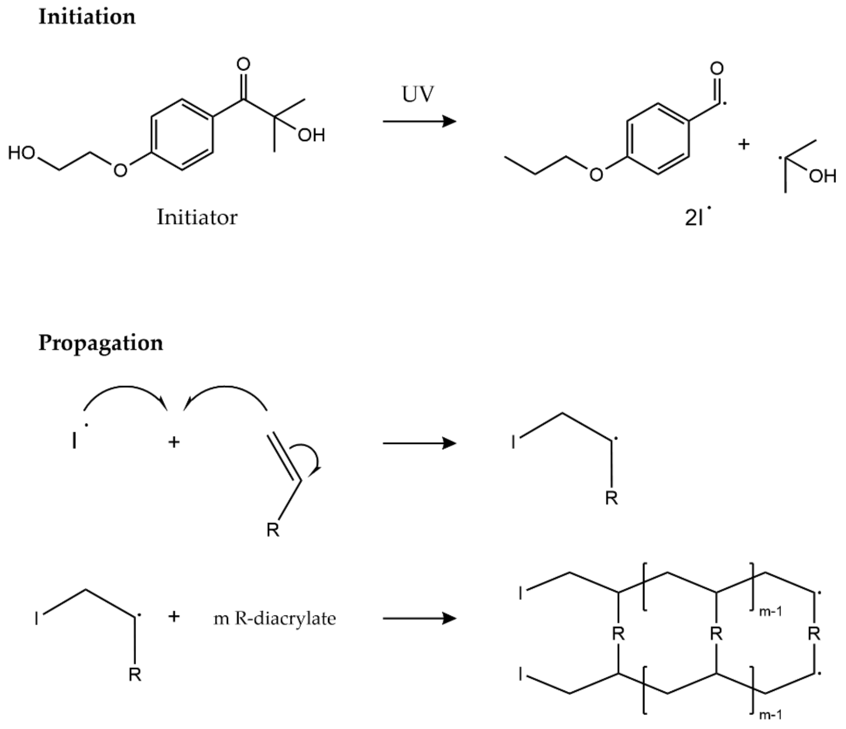

Figure S1: Schematic representation of UV-crosslinking for the generation of spherical hydrogel particles. Upon UV exposure, the initiator generates two radicals that will react with the acrylate functional groups present on either end of the PEG chain. This results in the crosslinking of multiple PEG chains together, forming a hydrogel.

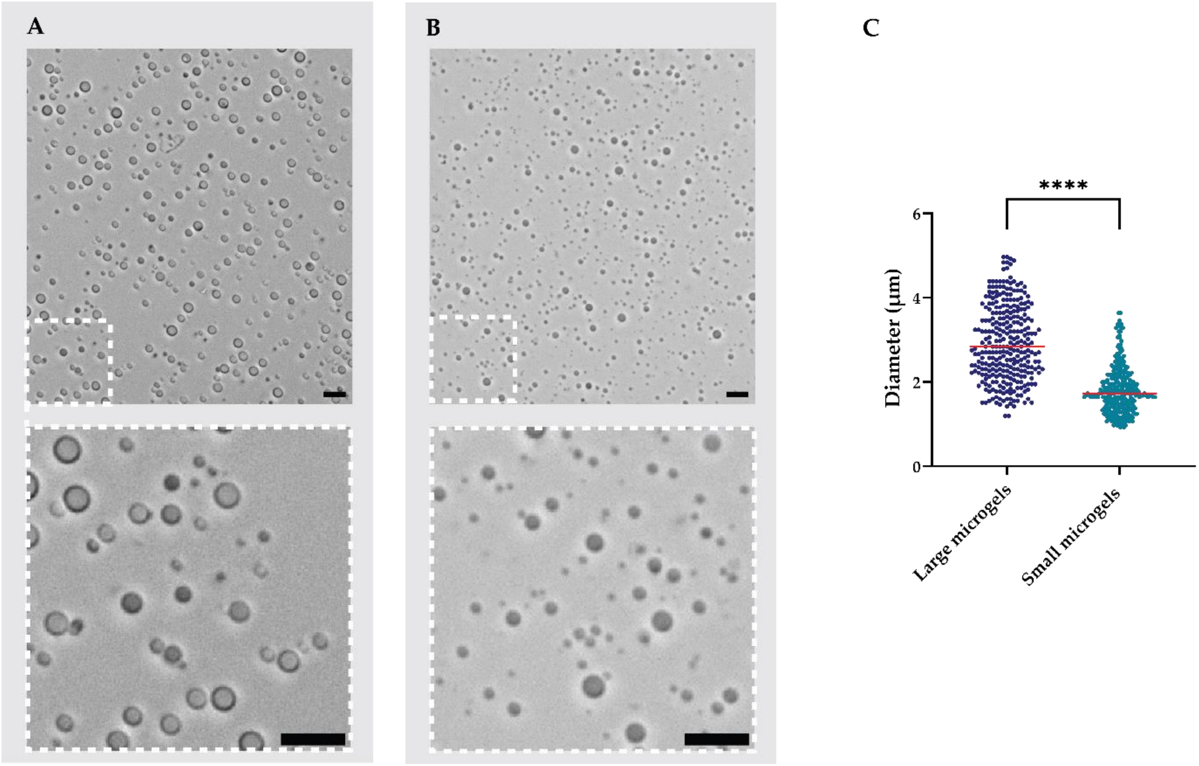

Figure S2. Centrifugation-based isolation of microgel fractions. A sample from Figure 2A (200:50 oil-to-polymer ratio) was centrifuged at 2,000 rpm for 5 min to sediment microgels and remove the nanohydrogel fraction. The pellet was resuspended in ultrapure water and centrifuged again at 2,000 rpm for 1 min to separate larger (A) and smaller (B) microgels. Scale bars, 10 µm. (C) Particle-size distributions of each fraction reveal a significant difference in mean size (Mann–Whitney test, $p < 0.0001$).

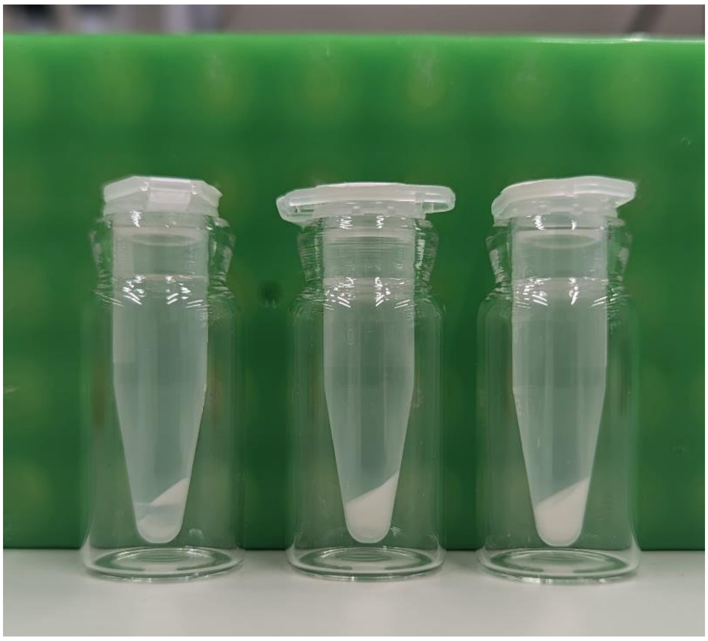

Figure S3. Centrifuged samples after vortexing at varying speeds (rpm). From left to right: 1200 rpm, 1600 rpm, and 2000 rpm. Complete emulsification is observed at 2000 rpm, while incomplete emulsification at 1200 rpm and 1600 rpm is indicated by transparent crosslinked hydrogel sediment at the bottom of the Eppendorf® tubes.

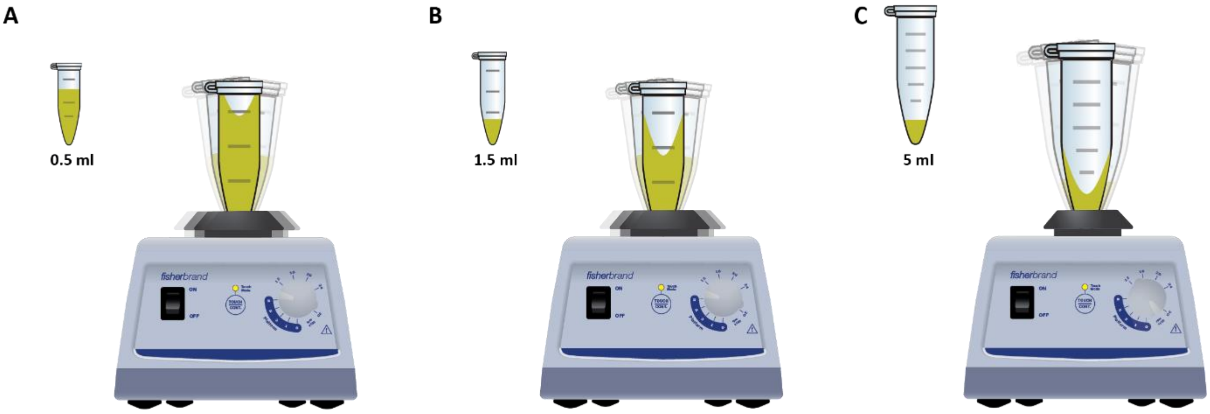

Figure S4. Schematic representation of the emulsion during vortexing in different Eppendorf® sizes. Sizes shown here are 0.5 ml (A), 1.5 ml (B) and 5 ml (C). The polymer-to-oil ratio was maintained consistently at 50:300 across all volumes. Larger container sizes facilitate a more uniform shear gradient distribution within the emulsion, promoting the formation of smaller hydrogel particles with reduced polydispersity.

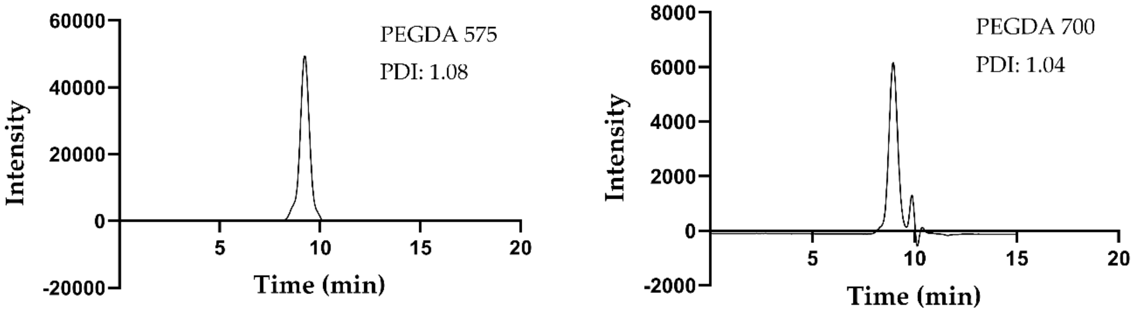

Figure S5. Gel permeation chromatography of PEGDA 575 (left) and 700 (right). PEGDA 700 shows improved PDI compared to 575, i.e. 1.04 and 1.08 respectively.

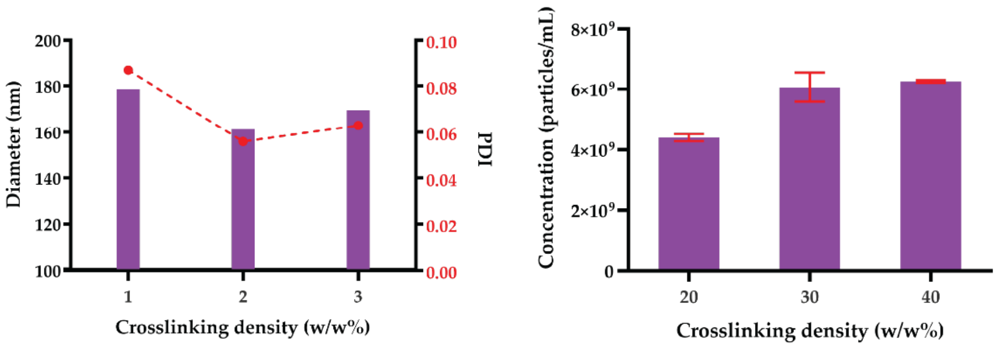

Figure S6. Effect of the crosslink density on the size and PDI. No clear trend was observed concerning the size or uniformity of the hydrogel population, but a clear increase in concentration was observed with increasing crosslink density.

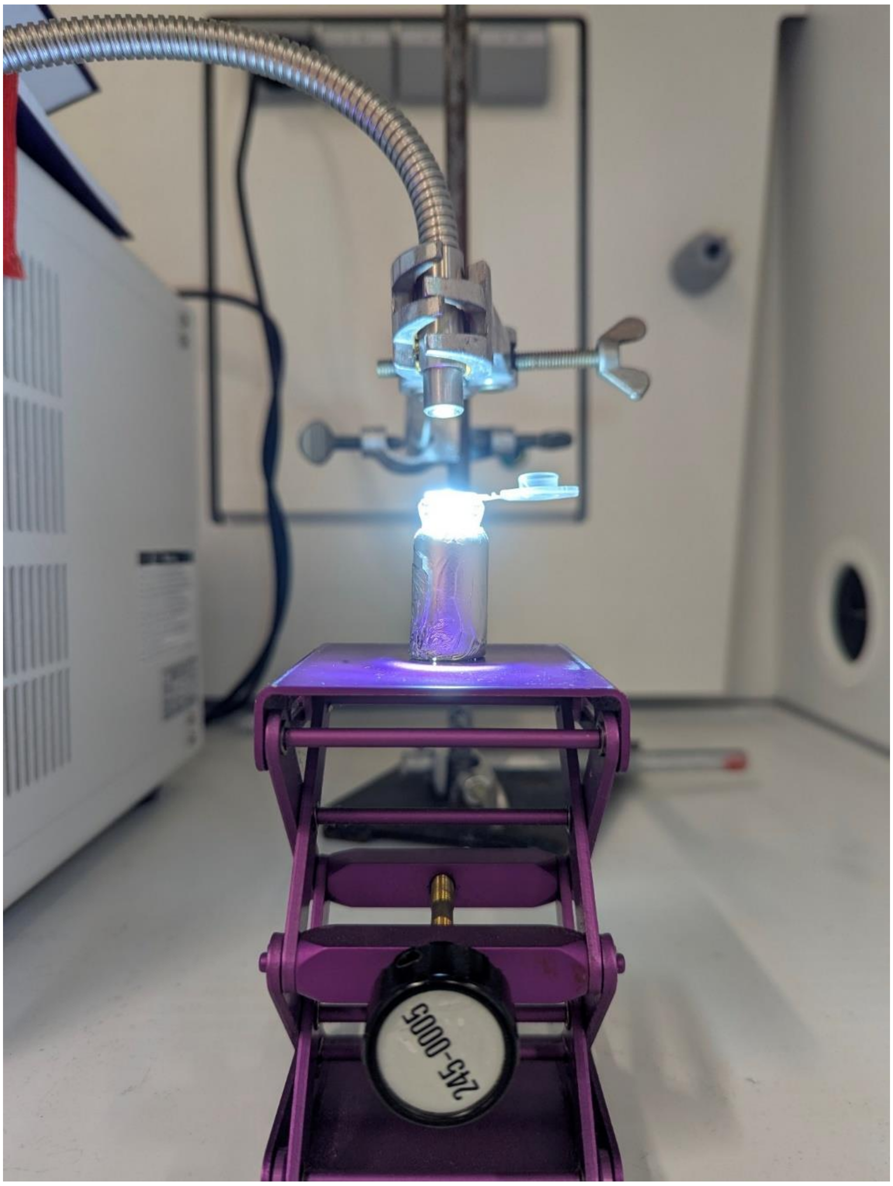

Figure S7. A representative image of the UV-polymerization setup is shown. To ensure reproducibility, the configuration, including the position and distance of the light source relative to the Eppendorf® tube, remained constant across all samples. The sample was placed upright in a glass vial wrapped in aluminum foil to reflect stray UV rays back onto the sample. The cap of the Eppendorf® tube was left open to avoid any UV-ray absorbance and to ensure optimal photopolymerization.

```
// 1) Ask for Labkit model
waitForUser("In the following window, please select Labkit Classifier file to use");
classifier_path = File.openDialog("Which labkit model to use?");

// 2) Ask for input and output folders
inputDir  = getDirectory("Choose input directory (with .tif files)");
outputDir = getDirectory("Choose output directory (for CSV results)");

// 3) Get all files in inputDir
fileList = getFileList(inputDir);

// 4) Process each .tif
for (i = 0; i < fileList.length; i++) {
    name = fileList[i];
    if (endsWith(name, ".tif") || endsWith(name, ".TIF")) {
        // Open image
        open(inputDir + name);

        // === your original processing ===
        // a) Labkit probability map
        run("Calculate Probability Map With Labkit",
            "segmenter_file=["+classifier_path+"] use_gpu=false");
        prob_title = getTitle();

        // b) Threshold & mask
        setThreshold(0.5, 1);
        run("Convert to Mask", "background=Dark create");
        mask_title = getTitle();
        close(prob_title);

        // c) Duplicate mask for Hough
        run("Duplicate...", "title=prob duplicate channels=1");
        close(mask_title);

        // d) Hough circle detection
        run("Hough Circle Transform",
            "minRadius=4, maxRadius=40, inc=1, minCircles=1, maxCircles=10000, "+
            "threshold=0.85, resolution=1000, ratio=1.0, bandwidth=10, "+
            "local_radius=10, reduce show_mask show_centroids results_table");

        // Wait here until the Results window exists
        while (!isOpen("Results")) {
            wait(500);
        }

        // === save results ===
        // Extract base name without extension
        base = replace(name, ".tif", "");
        base = replace(base, ".TIF", "");

        // Select and save the "Results" table as CSV
        selectWindow("Results");
        saveAs("Results", outputDir + base + "_results.csv");
        close("Results");

        // Close all images before next iteration
        run("Close All");
    }
}
```

Figure S8. Custom Fiji ImageJ v1.54f macro for micro hydrogel detection in brightfield images acquired on an Echo Revolution microscope.

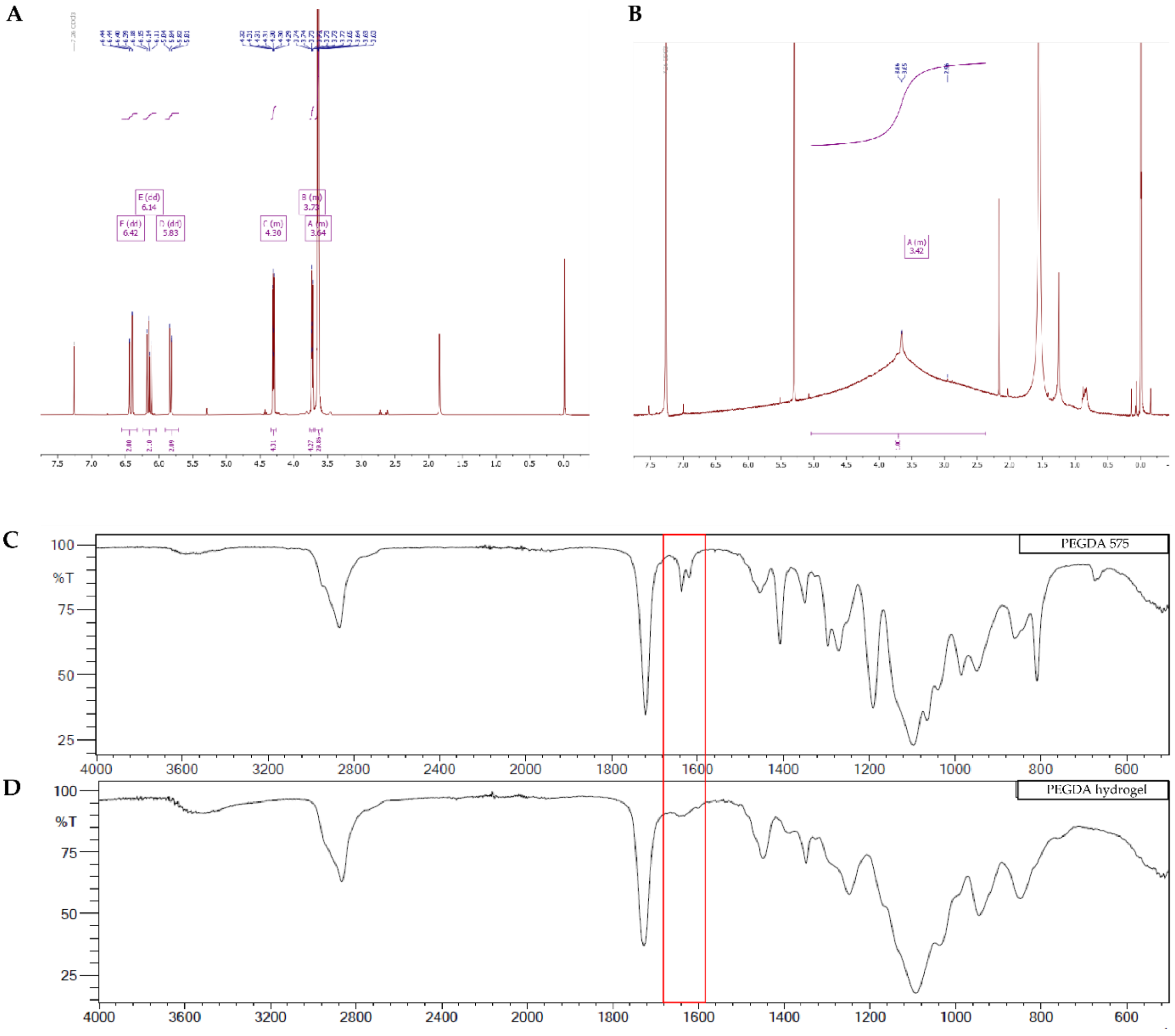

Figure S9. ^1H NMR and FTIR spectroscopy of the unpolymerized PEGDA (A, C) and the PEGDA hydrogel (B, D). Successful polymerization can be determined by the disappearance of the characteristic vinyl proton signals from the acrylate groups are observed at 6.42, 6.14, and 5.84 ppm, and the broadening of the PEG backbone peak at 3.64 ppm, typical for polymers in crosslinked gels. Additionally, the characteristic C=C stretching vibrations of the acrylate groups, observed between 1620–1680 cm^{-1}, are significantly reduced or absent after crosslinking.
